# 5-Iodo-3-phenyl-2,1-benzoxazole

**DOI:** 10.1107/S1600536813005862

**Published:** 2013-03-09

**Authors:** Yuriy Teslenko, Vasyl S. Matiychuk, Vasyl Kinzhybalo, Tadeusz Lis, Mykola D. Obushak

**Affiliations:** a Department of Organic Chemistry, Ivan Franko National University of Lviv, Kyryla and Mefodiya 6, Lviv 79005, Ukraine; b Institute of Low Temperature and Structure Research, Okolna 2, 50-422 Wrocław, Poland; c Faculty of Chemistry, University of Wrocław, 14 Joliot-Curie St, 50-383 Wrocław, Poland

## Abstract

The title compound, C_13_H_8_INO, was prepared by a condensation reaction of 4-nitro­benzene with phenyl­acetonitrile in NaOH–ethanol solution. There are two independent mol­ecules in the asymmetric unit, in which the dihedral angles between the benzene ring and the benzoisoxazole unit are 4.2 (3) and 4.1 (3)°. The crystal packing is governed by C—H⋯N, C—I⋯π and C—I⋯O inter­actions.

## Related literature

For the biologial activity and applications of benzo[*c*]isoxazoles, see: McEvoy *et al.* (1968 ▶); Hester *et al.* (1989 ▶); Walsh *et al.* (1990 ▶); Angibaud *et al.* (2003 ▶). For a related structure, see: Teslenko *et al.* (2008 ▶). For a general synthetic procedure, see: Davis & Pizzini (1960 ▶).
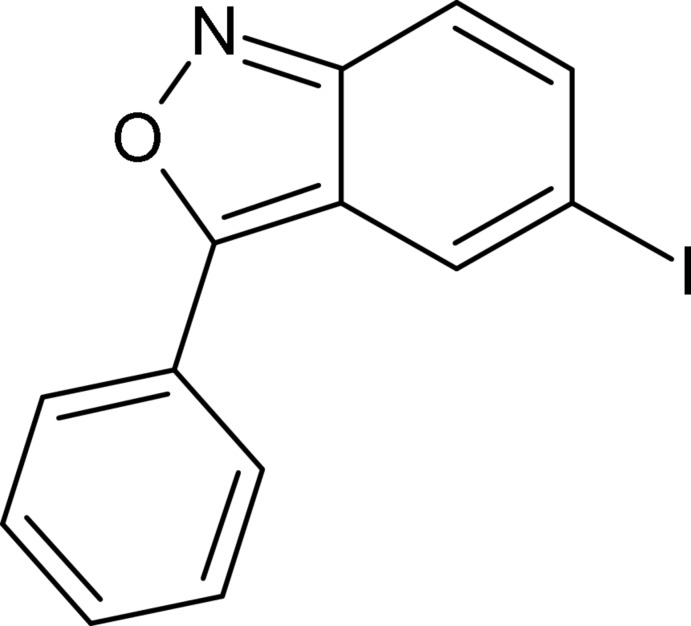


## Experimental

### 

#### Crystal data

C_13_H_8_INO
*M* *_r_* = 321.10
Monoclinic, 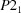

*a* = 5.381 (3) Å
*b* = 15.225 (7) Å
*c* = 13.749 (7) Å
β = 94.92 (3)°
*V* = 1122.2 (10) Å^3^
*Z* = 4
Mo *K*α radiation
μ = 2.83 mm^−1^
*T* = 100 K
0.25 × 0.08 × 0.03 mm

#### Data collection

Kuma KM-4-CCD four-circle diffractometer
Absorption correction: analytical (*CrysAlis RED*; Oxford Diffraction, 2006 ▶) *T*_min_ = 0.44, *T*_max_ = 0.80
15060 measured reflections
6015 independent reflections
4621 reflections with *I* > 2σ(*I*)
*R*_int_ = 0.053

#### Refinement

*R*[*F* ^2^ > 2σ(*F* ^2^)] = 0.041
*wR*(*F* ^2^) = 0.090
*S* = 1.00
6015 reflections
289 parameters
1 restraint
H-atom parameters constrained
Δρ_max_ = 1.98 e Å^−3^
Δρ_min_ = −1.01 e Å^−3^
Absolute structure: Flack (1983 ▶), 1659 Friedel pairs
Flack parameter: 0.00 (3)

### 

Data collection: *CrysAlis CCD* (Oxford Diffraction, 2006 ▶); cell refinement: *CrysAlis RED* (Oxford Diffraction, 2006 ▶); data reduction: *CrysAlis RED*; program(s) used to solve structure: *SHELXS97* (Sheldrick, 2008 ▶); program(s) used to refine structure: *SHELXL97* (Sheldrick, 2008 ▶); molecular graphics: *DIAMOND* (Brandenburg, 2006 ▶); software used to prepare material for publication: *publCIF* (Westrip, 2010 ▶).

## Supplementary Material

Crystal structure: contains datablock(s) I, global. DOI: 10.1107/S1600536813005862/gk2554sup1.cif

Structure factors: contains datablock(s) I. DOI: 10.1107/S1600536813005862/gk2554Isup2.hkl

Supplementary material file. DOI: 10.1107/S1600536813005862/gk2554Isup3.cdx

Supplementary material file. DOI: 10.1107/S1600536813005862/gk2554Isup4.cml

Additional supplementary materials:  crystallographic information; 3D view; checkCIF report

## Figures and Tables

**Table 1:** Intermolecular interactions (Å, °) *Cg* is the centroid of the C1*B*–C6*B* ring.

*D*—H⋯*A*	*D*—H	H⋯*A*	*D*⋯*A*	*D*—H⋯*A*
C3*A*—H3*A*⋯N1*B* ^i^	0.95	2.40	3.247 (7)	149
C11*A*—H11*A*⋯N1*A* ^ii^	0.95	2.47	3.339 (8)	152
C4*A*—I1*A*⋯*Cg* ^iii^	2.100 (5)	3.618 (2)	5.637 (6)	160.0 (2)
C4*B*—I1*B*⋯O1*A*	2.100 (5)	3.335 (5)	5.325 (7)	156.3 (2)

Symmetry codes: (i) 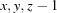; (ii) 
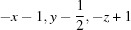
; (iii) 
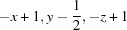
.

**Fig. 1.**
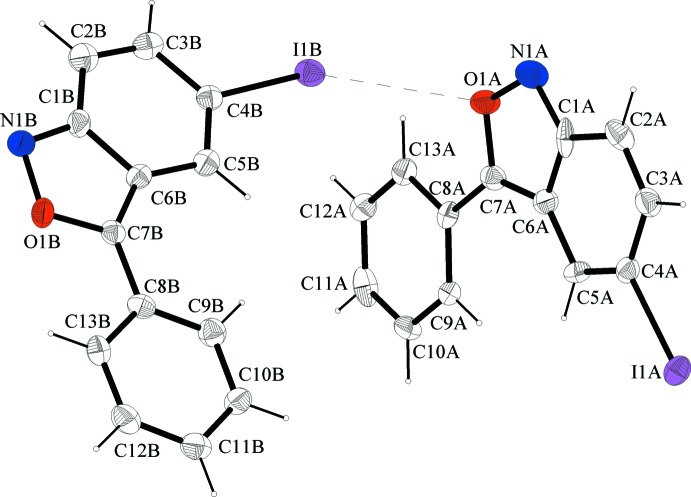
The asymmetric unit of the title compound with atom labeling scheme. The displacement ellipsoids are drawn at the 50% probability level.

**Fig. 2.**
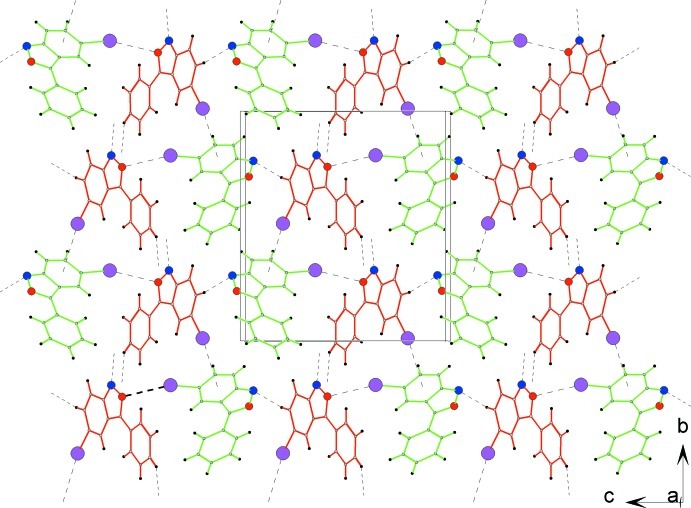
The crystal packing of the title compound showing intermolecular interactions as dashed lines (molecule A - red, molecule B - green).

## supplementary crystallographic information

### Comment

Our interest in benzo[*c*]isoxazoles is concerned with their application as precursors of a variety of bioactive compounds (Angibaud *et al.*, 2003; Walsh *et al.*, 1990; Hester *et al.*, 1989; McEvoy *et al.*, 1968). The title compound will be used in our further investigations as arylation agent in palladium-catalyzed reactions with alkenes and alkynes.

The title compound crystalizes in the noncentrosymmetric monoclinic *P*2_1_ space group with two independent molecules in the asymmetric part (A and B), see Fig. 1. The molecules are almost planar, the dihedral angles between the mean planes of benzoisoxazole and benzene rings being 4.2 (3)° and 4.1 (3)° for A and B, respectively. The geometrical parameters of the molecules are similar and consistent with the previously studied 2,1-benzoxazole derivatives (Teslenko *et al.*, 2008).

Crystal packing is governed by hydrogen bonds of C–H···N type and other intermolecular interactions including C–I···π and C–I···O. Intermolecular interactions C4A–I1A···*C_g_^iii^* (C_g_ is a centroid of C1B/C6B aromatic ring) and C4B–I1B···O1A connect the molecules into chains propagating in *b*-axis direction along 2_1_ screw axis (see Fig. 2). Hydrogen bond C3A–H3A···N1B*^i^* connects the chains into corrugated layer parallel to the *bc*-plane. Hydrogen bond C11A–H11A···N1A*^ii^* binds successive layers.

### Experimental

Phenylacetonitrile (1.4 g, 12 mmol) and 5 ml of benzene solution of 4-iodonitrobenene (2.49 g, 10 mmol) were added with stirring to 40 ml of ethanol solution of potassium hydroxide (4 g, 0.1 mole). The mixture was stirred for 4 h at 323 K, then poured into 150 ml of water and acidified with hydrochloric acid. The precipitate was isolated by filtration, washed with water and dried. Recrystallization of crude product from ethanol gave 2.57 g (80% yield) of 5-iodo-3-phenyl-2,1-benzoxazole as pale yellow needles suitable for X-ray analysis, m.p. 390–391 K.

### Refinement

All H atoms were found in difference Fourier maps. All H atoms were positioned geometrically and treated as riding on their carriers, with C–H = 0.95 Å and *U*_iso_(H) = values of 1.2*U*_eq_(C).

### Crystal data

**Table:**

C_13_H_8_INO	*F*(000) = 616
*M**_r_* = 321.10	*D*_x_ = 1.901 Mg m^−^^3^
Monoclinic, *P*2_1_	Melting point = 390–391 K
Hall symbol: P 2yb	Mo *K*α radiation, λ = 0.71073 Å
*a* = 5.381 (3) Å	Cell parameters from 15060 reflections
*b* = 15.225 (7) Å	θ = 3.0–34.7°
*c* = 13.749 (7) Å	µ = 2.83 mm^−^^1^
β = 94.92 (3)°	*T* = 100 K
*V* = 1122.2 (10) Å^3^	Needle, pale yellow
*Z* = 4	0.25 × 0.08 × 0.03 mm

### Data collection

**Table:**

Kuma KM-4-CCD four-circle diffractometer	6015 independent reflections
Radiation source: fine-focus sealed tube	4621 reflections with *I* > 2σ(*I*)
Graphite monochromator	*R*_int_ = 0.053
ω scans	θ_max_ = 34.7°, θ_min_ = 3.0°
Absorption correction: analytical (*CrysAlis RED*; Oxford Diffraction, 2006)	*h* = −8→7
*T*_min_ = 0.44, *T*_max_ = 0.80	*k* = −17→23
15060 measured reflections	*l* = −20→21

### Refinement

**Table:**

Refinement on *F*^2^	Secondary atom site location: difference Fourier map
Least-squares matrix: full	Hydrogen site location: inferred from neighbouring sites
*R*[*F*^2^ > 2σ(*F*^2^)] = 0.041	H-atom parameters constrained
*wR*(*F*^2^) = 0.090	*w* = 1/[σ^2^(*F*_o_^2^) + (0.046*P*)^2^] where *P* = (*F*_o_^2^ + 2*F*_c_^2^)/3
*S* = 1.00	(Δ/σ)_max_ = 0.001
6015 reflections	Δρ_max_ = 1.98 e Å^−^^3^
289 parameters	Δρ_min_ = −1.01 e Å^−^^3^
1 restraint	Absolute structure: Flack (1983), 1659 Friedel pairs
Primary atom site location: structure-invariant direct methods	Flack parameter: 0.00 (3)

### Special details

**Table:**

Geometry. All e.s.d.'s (except the e.s.d. in the dihedral angle between two l.s. planes) are estimated using the full covariance matrix. The cell e.s.d.'s are taken into account individually in the estimation of e.s.d.'s in distances, angles and torsion angles; correlations between e.s.d.'s in cell parameters are only used when they are defined by crystal symmetry. An approximate (isotropic) treatment of cell e.s.d.'s is used for estimating e.s.d.'s involving l.s. planes.
Refinement. Refinement of *F*^2^ against ALL reflections. The weighted *R*-factor *wR* and goodness of fit *S* are based on *F*^2^, conventional *R*-factors *R* are based on *F*, with *F* set to zero for negative *F*^2^. The threshold expression of *F*^2^ > σ(*F*^2^) is used only for calculating *R*-factors(gt) *etc*. and is not relevant to the choice of reflections for refinement. *R*-factors based on *F*^2^ are statistically about twice as large as those based on *F*, and *R*- factors based on ALL data will be even larger.

### Fractional atomic coordinates and isotropic or equivalent isotropic displacement parameters (Å^2^)

**Table:**

	*x*	*y*	*z*	*U*_iso_*/*U*_eq_	
I1A	0.51472 (6)	0.01154 (2)	0.19512 (2)	0.02514 (9)	
O1A	−0.2353 (8)	0.2574 (3)	0.4305 (3)	0.0262 (9)	
N1A	−0.0803 (9)	0.3076 (4)	0.3739 (4)	0.0316 (10)	
C1A	0.0530 (10)	0.2453 (4)	0.3322 (4)	0.0256 (12)	
C2A	0.2425 (11)	0.2686 (4)	0.2666 (5)	0.0303 (13)	
H2A	0.2765	0.3276	0.2499	0.036*	
C3A	0.3651 (11)	0.2002 (4)	0.2316 (4)	0.0261 (11)	
H3A	0.4926	0.2110	0.1894	0.031*	
C4A	0.3088 (10)	0.1115 (4)	0.2561 (4)	0.0219 (10)	
C5A	0.1314 (10)	0.0903 (4)	0.3171 (4)	0.0204 (10)	
H5A	0.0981	0.0309	0.3328	0.024*	
C6A	−0.0029 (10)	0.1613 (3)	0.3564 (4)	0.0201 (10)	
C7A	−0.1871 (10)	0.1707 (4)	0.4203 (4)	0.0213 (10)	
C8A	−0.3335 (10)	0.1101 (4)	0.4753 (4)	0.0204 (10)	
C9A	−0.2895 (11)	0.0191 (4)	0.4735 (4)	0.0260 (11)	
H9A	−0.1608	−0.0034	0.4373	0.031*	
C10A	−0.4324 (12)	−0.0380 (4)	0.5240 (4)	0.0267 (12)	
H10A	−0.3982	−0.0992	0.5230	0.032*	
C11A	−0.6211 (10)	−0.0080 (4)	0.5750 (4)	0.0296 (14)	
H11A	−0.7201	−0.0478	0.6084	0.036*	
C12A	−0.6676 (11)	0.0838 (4)	0.5776 (4)	0.0229 (11)	
H12A	−0.7973	0.1054	0.6138	0.027*	
C13A	−0.5264 (10)	0.1421 (4)	0.5282 (4)	0.0220 (11)	
H13A	−0.5597	0.2033	0.5300	0.026*	
I1B	−0.05111 (7)	0.30782 (2)	0.66158 (3)	0.02711 (9)	
O1B	0.7816 (8)	0.2167 (3)	1.0229 (3)	0.0258 (8)	
N1B	0.6242 (9)	0.2844 (3)	1.0482 (4)	0.0270 (10)	
C1B	0.4622 (10)	0.2937 (3)	0.9704 (4)	0.0232 (11)	
C2B	0.2531 (11)	0.3519 (4)	0.9611 (5)	0.0272 (12)	
H2B	0.2166	0.3890	1.0136	0.033*	
C3B	0.1074 (11)	0.3530 (4)	0.8749 (4)	0.0249 (11)	
H3B	−0.0335	0.3907	0.8673	0.030*	
C4B	0.1657 (9)	0.2974 (3)	0.7956 (4)	0.0206 (10)	
C5B	0.3589 (10)	0.2384 (4)	0.8030 (4)	0.0220 (11)	
H5B	0.3902	0.2010	0.7501	0.026*	
C6B	0.5112 (10)	0.2353 (3)	0.8931 (4)	0.0199 (10)	
C7B	0.7150 (10)	0.1873 (3)	0.9304 (4)	0.0196 (10)	
C8B	0.8618 (10)	0.1135 (4)	0.8959 (4)	0.0216 (10)	
C9B	0.8005 (11)	0.0769 (4)	0.8031 (4)	0.0258 (12)	
H9B	0.6644	0.0996	0.7621	0.031*	
C10B	0.9389 (10)	0.0082 (4)	0.7720 (4)	0.0255 (10)	
H10B	0.8969	−0.0158	0.7089	0.031*	
C11B	1.1383 (12)	−0.0274 (4)	0.8297 (4)	0.0285 (12)	
H11B	1.2315	−0.0753	0.8076	0.034*	
C12B	1.1968 (11)	0.0099 (5)	0.9218 (4)	0.0301 (11)	
H12B	1.3328	−0.0131	0.9626	0.036*	
C13B	1.0630 (11)	0.0790 (4)	0.9549 (4)	0.0253 (11)	
H13B	1.1071	0.1032	1.0177	0.030*	

### Atomic displacement parameters (Å^2^)

**Table:**

	*U*^11^	*U*^22^	*U*^33^	*U*^12^	*U*^13^	*U*^23^
I1A	0.02109 (16)	0.03002 (19)	0.02445 (17)	0.00079 (15)	0.00275 (13)	−0.00571 (15)
O1A	0.028 (2)	0.021 (2)	0.031 (2)	0.0056 (16)	0.0085 (17)	0.0011 (16)
N1A	0.036 (3)	0.024 (2)	0.037 (3)	−0.001 (3)	0.014 (2)	0.007 (2)
C1A	0.010 (2)	0.055 (4)	0.011 (2)	0.000 (2)	−0.0008 (18)	0.000 (2)
C2A	0.029 (3)	0.033 (3)	0.030 (3)	−0.001 (3)	0.006 (2)	0.011 (2)
C3A	0.029 (3)	0.028 (3)	0.022 (3)	0.001 (2)	0.006 (2)	0.004 (2)
C4A	0.021 (3)	0.025 (3)	0.020 (2)	0.006 (2)	−0.0003 (19)	−0.002 (2)
C5A	0.021 (3)	0.015 (2)	0.025 (3)	0.000 (2)	0.001 (2)	−0.0027 (19)
C6A	0.021 (3)	0.018 (3)	0.021 (3)	0.000 (2)	0.001 (2)	0.0002 (19)
C7A	0.020 (3)	0.021 (3)	0.022 (3)	0.001 (2)	−0.003 (2)	0.0008 (19)
C8A	0.021 (2)	0.023 (3)	0.017 (2)	0.001 (2)	0.0032 (19)	−0.0026 (19)
C9A	0.042 (3)	0.018 (3)	0.019 (2)	0.005 (3)	0.005 (2)	−0.001 (2)
C10A	0.039 (3)	0.017 (3)	0.024 (3)	−0.001 (2)	0.000 (2)	0.002 (2)
C11A	0.021 (3)	0.048 (4)	0.020 (3)	−0.012 (2)	0.001 (2)	0.008 (2)
C12A	0.020 (3)	0.026 (3)	0.023 (3)	0.000 (2)	0.000 (2)	−0.003 (2)
C13A	0.020 (3)	0.025 (3)	0.021 (3)	0.003 (2)	0.001 (2)	−0.002 (2)
I1B	0.02550 (18)	0.02499 (18)	0.03013 (19)	0.00099 (17)	−0.00173 (14)	0.00057 (16)
O1B	0.032 (2)	0.028 (2)	0.0171 (19)	−0.0014 (18)	0.0017 (15)	−0.0014 (16)
N1B	0.035 (3)	0.021 (2)	0.025 (2)	−0.0026 (19)	0.004 (2)	0.0006 (17)
C1B	0.030 (3)	0.022 (3)	0.019 (2)	−0.005 (2)	0.007 (2)	0.0006 (18)
C2B	0.027 (3)	0.026 (3)	0.030 (3)	−0.003 (2)	0.012 (2)	0.001 (2)
C3B	0.021 (3)	0.024 (3)	0.032 (3)	0.000 (2)	0.009 (2)	−0.001 (2)
C4B	0.022 (2)	0.018 (3)	0.022 (2)	−0.004 (2)	0.0011 (19)	0.0015 (18)
C5B	0.023 (3)	0.021 (3)	0.022 (3)	−0.004 (2)	0.003 (2)	−0.0012 (19)
C6B	0.021 (3)	0.018 (2)	0.021 (3)	−0.003 (2)	0.005 (2)	−0.0020 (18)
C7B	0.023 (3)	0.017 (2)	0.019 (2)	−0.005 (2)	0.002 (2)	0.0018 (18)
C8B	0.020 (3)	0.019 (2)	0.026 (3)	−0.003 (2)	0.003 (2)	0.003 (2)
C9B	0.024 (3)	0.028 (3)	0.025 (3)	0.000 (2)	0.000 (2)	0.001 (2)
C10B	0.026 (3)	0.025 (3)	0.026 (2)	0.002 (3)	0.0045 (19)	−0.004 (2)
C11B	0.029 (3)	0.026 (3)	0.031 (3)	0.003 (2)	0.009 (2)	0.004 (2)
C12B	0.029 (3)	0.031 (3)	0.029 (3)	0.005 (3)	−0.003 (2)	0.006 (3)
C13B	0.027 (3)	0.028 (3)	0.020 (3)	0.001 (2)	−0.002 (2)	0.002 (2)

### Geometric parameters (Å, º)

**Table:**

I1A—C4A	2.100 (5)	I1B—C4B	2.100 (5)
O1A—C7A	1.354 (6)	O1B—C7B	1.366 (6)
O1A—N1A	1.413 (6)	O1B—N1B	1.397 (6)
N1A—C1A	1.346 (8)	N1B—C1B	1.329 (8)
C1A—C6A	1.361 (8)	C1B—C6B	1.427 (7)
C1A—C2A	1.462 (8)	C1B—C2B	1.430 (8)
C2A—C3A	1.344 (9)	C2B—C3B	1.363 (9)
C2A—H2A	0.9500	C2B—H2B	0.9500
C3A—C4A	1.430 (8)	C3B—C4B	1.437 (8)
C3A—H3A	0.9500	C3B—H3B	0.9500
C4A—C5A	1.362 (7)	C4B—C5B	1.371 (8)
C5A—C6A	1.432 (7)	C5B—C6B	1.426 (8)
C5A—H5A	0.9500	C5B—H5B	0.9500
C6A—C7A	1.387 (7)	C6B—C7B	1.379 (7)
C7A—C8A	1.466 (7)	C7B—C8B	1.475 (8)
C8A—C9A	1.405 (8)	C8B—C13B	1.398 (8)
C8A—C13A	1.405 (7)	C8B—C9B	1.406 (8)
C9A—C10A	1.387 (8)	C9B—C10B	1.374 (8)
C9A—H9A	0.9500	C9B—H9B	0.9500
C10A—C11A	1.362 (8)	C10B—C11B	1.389 (8)
C10A—H10A	0.9500	C10B—H10B	0.9500
C11A—C12A	1.420 (9)	C11B—C12B	1.399 (9)
C11A—H11A	0.9500	C11B—H11B	0.9500
C12A—C13A	1.384 (8)	C12B—C13B	1.374 (9)
C12A—H12A	0.9500	C12B—H12B	0.9500
C13A—H13A	0.9500	C13B—H13B	0.9500
			
C7A—O1A—N1A	110.0 (4)	C7B—O1B—N1B	110.9 (4)
C1A—N1A—O1A	102.4 (5)	C1B—N1B—O1B	104.3 (4)
N1A—C1A—C6A	114.9 (5)	N1B—C1B—C6B	112.5 (5)
N1A—C1A—C2A	121.2 (6)	N1B—C1B—C2B	126.5 (5)
C6A—C1A—C2A	123.9 (6)	C6B—C1B—C2B	121.0 (5)
C3A—C2A—C1A	115.1 (6)	C3B—C2B—C1B	118.3 (5)
C3A—C2A—H2A	122.5	C3B—C2B—H2B	120.8
C1A—C2A—H2A	122.5	C1B—C2B—H2B	120.8
C2A—C3A—C4A	121.7 (5)	C2B—C3B—C4B	120.4 (5)
C2A—C3A—H3A	119.1	C2B—C3B—H3B	119.8
C4A—C3A—H3A	119.1	C4B—C3B—H3B	119.8
C5A—C4A—C3A	122.9 (5)	C5B—C4B—C3B	122.9 (5)
C5A—C4A—I1A	119.7 (4)	C5B—C4B—I1B	118.4 (4)
C3A—C4A—I1A	117.4 (4)	C3B—C4B—I1B	118.7 (4)
C4A—C5A—C6A	117.1 (5)	C4B—C5B—C6B	117.5 (5)
C4A—C5A—H5A	121.4	C4B—C5B—H5B	121.2
C6A—C5A—H5A	121.4	C6B—C5B—H5B	121.2
C1A—C6A—C7A	104.0 (5)	C7B—C6B—C5B	136.0 (5)
C1A—C6A—C5A	119.2 (5)	C7B—C6B—C1B	104.2 (5)
C7A—C6A—C5A	136.8 (5)	C5B—C6B—C1B	119.8 (5)
O1A—C7A—C6A	108.7 (5)	O1B—C7B—C6B	108.0 (4)
O1A—C7A—C8A	116.4 (5)	O1B—C7B—C8B	116.3 (5)
C6A—C7A—C8A	134.9 (5)	C6B—C7B—C8B	135.6 (5)
C9A—C8A—C13A	119.0 (5)	C13B—C8B—C9B	119.2 (5)
C9A—C8A—C7A	120.9 (5)	C13B—C8B—C7B	120.6 (5)
C13A—C8A—C7A	120.1 (5)	C9B—C8B—C7B	120.2 (5)
C10A—C9A—C8A	120.5 (5)	C10B—C9B—C8B	119.6 (6)
C10A—C9A—H9A	119.8	C10B—C9B—H9B	120.2
C8A—C9A—H9A	119.8	C8B—C9B—H9B	120.2
C11A—C10A—C9A	121.2 (5)	C9B—C10B—C11B	122.1 (6)
C11A—C10A—H10A	119.4	C9B—C10B—H10B	118.9
C9A—C10A—H10A	119.4	C11B—C10B—H10B	118.9
C10A—C11A—C12A	118.9 (5)	C10B—C11B—C12B	117.5 (6)
C10A—C11A—H11A	120.5	C10B—C11B—H11B	121.3
C12A—C11A—H11A	120.5	C12B—C11B—H11B	121.3
C13A—C12A—C11A	120.9 (5)	C13B—C12B—C11B	121.8 (5)
C13A—C12A—H12A	119.6	C13B—C12B—H12B	119.1
C11A—C12A—H12A	119.6	C11B—C12B—H12B	119.1
C12A—C13A—C8A	119.5 (5)	C12B—C13B—C8B	119.8 (5)
C12A—C13A—H13A	120.3	C12B—C13B—H13B	120.1
C8A—C13A—H13A	120.3	C8B—C13B—H13B	120.1

### Hydrogen-bond geometry (Å, º)

Cg is the centroid of the C1B–C6B ring.

**Table:**

*D*—H···*A*	*D*—H	H···*A*	*D*···*A*	*D*—H···*A*
C3*A*—H3*A*···N1*B*^i^	0.95	2.40	3.247 (7)	149
C11*A*—H11*A*···N1*A*^ii^	0.95	2.47	3.339 (8)	152
C4*A*—I1*A*···*Cg*^iii^	2.10 (1)	3.62 (1)	5.637 (6)	160 (1)
C4*B*—I1*B*···O1*A*	2.10 (1)	3.34 (1)	5.325 (7)	156 (1)

Symmetry codes: (i) *x*, *y*, *z*−1; (ii) −*x*−1, *y*−1/2, −*z*+1; (iii) −*x*+1, *y*−1/2, −*z*+1.

## References

[bb1] Angibaud, P., Bourdrez, X., Devine, A., End, D. W., Freyne, E., Ligny, Y., Muller, P., Mannens, G., Pilatte, I., Poncelet, V., Skrzat, S., Smets, G., Van Dun, J., Van Remoortere, P., Venet, M. & Wouters, W. (2003). *Bioorg. Med. Chem. Lett.* **13**, 1543–1548. 10.1016/s0960-894x(03)00180-x 12699751

[bb2] Brandenburg, K. (2006). *DIAMOND* Crystal Impact GbR, Bonn, Germany.

[bb3] Davis, R. B. & Pizzini, L. C. (1960). *J. Org. Chem.* **25**, 1884–1888.

[bb4] Flack, H. D. (1983). *Acta Cryst.* A**39**, 876–881.

[bb5] Hester, J. B., Ludens, J. H., Emmert, D. E. & West, B. E. (1989). *J. Med. Chem.* **32**, 1157–1163. 10.1021/jm00126a003 2724291

[bb6] McEvoy, F. J., Greenblatt, E. N., Osterrerg, A. C. & Allen, G. R. Jr (1968). *J. Med. Chem.* **11**, 1248–1250. 10.1021/jm00312a602 5680056

[bb7] Oxford Diffraction (2006). *CrysAlis CCD* and *CrysAlis RED* Oxford Diffraction Ltd, Wrocław, Poland.

[bb8] Sheldrick, G. M. (2008). *Acta Cryst.* A**64**, 112–122. 10.1107/S0108767307043930 18156677

[bb9] Teslenko, Y., Matiychuk, V., Obushak, M., Kinzhybalo, V. & Ślepokura, K. (2008). *Acta Cryst.* E**64**, o2420. 10.1107/S1600536808037872 PMC2959907 21581388

[bb10] Walsh, D. A., Moran, H. W., Shamblee, D. A. & Welstead, W. J. (1990). *J. Med. Chem.* **33**, 2296–2304. 10.1021/jm00170a039 2115589

[bb11] Westrip, S. P. (2010). *J. Appl. Cryst.* **43**, 920–925.

